# Mutational analyses of an instability domain reveal its conserved role in the regulation of class-B ARF levels in *Arabidopsis*

**DOI:** 10.1073/pnas.2537963123

**Published:** 2026-06-03

**Authors:** Zhaonan Ban, Michael J. Prigge, Yinglin Zhu, Nicholas Morffy, Wesley R. Neher, Andrew Muroyama, Lucia Strader, Mark Estelle

**Affiliations:** a Department of Cell and Developmental Biology, School of Biological Sciences, University of California, San Diego, La Jolla, CA 92093; b Department of Biology, Duke University, Durham, NC 27708; c Salk Institute for Biological Sciences, La Jolla, CA 92037

**Keywords:** auxin, ARF, plant development

## Abstract

The plant hormone auxin is a central regulator of plant growth and development. The canonical nuclear auxin signaling pathway acts through the regulation of gene transcription, and the protein levels of core component AUXIN RESPONSE FACTORS (ARFs) are key to this regulation. Here, we investigate the physiological role of a conserved instability (INS) domain in two class-B ARFs, ARF2 and ARF3, in *Arabidopsis thaliana*. Using native-promoter transgenic lines expressing different ARF2 variants, we show that T298N, T298D, and T298E mutant versions of the INS domain cause pronounced auxin-related phenotypes, including male sterility and defective root hair development. Notably, the T298D and T298E ARF2 variants, which exhibit increased protein stability, persist in epidermal nuclei within the root differentiation zone and are associated with shorter, branched root hairs, whereas wild-type ARF2 levels decline in this region coincident with root hair elongation. These lines also display auxin-resistant primary root elongation and lateral root promotion, along with reduced DR5:Luciferase responses, correlating with impaired ubiquitylation and enhanced ARF2 stability. Similarly, stabilized ARF3 variants (S293E and S293N) exhibit auxin resistance and severe developmental defects, indicating a shared regulatory mechanism among class-B ARFs. Our findings highlight critical physiological roles for class-B ARFs and verified the conserved function of the instability domain in controlling protein stability in *Arabidopsis*. We also reveal that INS domain-mediated turnover of ARF2 restricts their spatial accumulation and is essential for root hair elongation, providing insight into how signaling specificity is achieved through posttranslational control of ARF activity.

Auxin is a major plant hormone regulating diverse physiological processes vital for plant growth and development. The canonical auxin signaling pathway modulates gene expression through the SCF^TIR1/AFB^-Aux/IAA-ARF nuclear module. At low auxin levels, the Aux/IAA (INDOLE ACETIC ACID–INDUCED PROTEIN) transcriptional repressors bind to AUXIN RESPONSE FACTORS (ARFs) and recruit the corepressors TOPLESS (TPL), preventing ARF-mediated activation of auxin-responsive genes. Elevated auxin levels promote the binding of TIR1/AFBs (TRANSPORT INHIBITOR RESPONSE/AUXIN SIGNALING F-BOX) coreceptors to the Aux/IAAs, facilitating their ubiquitylation and subsequent degradation. This degradation relieves ARFs from repression, thereby activating downstream gene expression (1, 2).

In land plants, ARFs are typically classified into three subclasses: class-A, -B, and -C (3, 4). A typical ARF protein contains an N-terminal B3-type DNA binding domain (DBD) that recognizes auxin-response elements, a middle region that dictates activator or repressor function, and a C-terminal PB1 domain (formerly called III and IV) that mediate protein–protein interactions through dimerization with Aux/IAAs and other ARFs (4–6). In the auxin signaling pathway, class-A ARFs are generally considered as transcriptional activators, whereas some class-B and -C ARFs have been shown to function as repressors (5, 7, 8).

In *Arabidopsis*, ARFs are encoded by a large gene family consisting of 22 members: 5 class-A, 14 class-B, and 3 class-C ARFs. While class-A ARFs have been well studied and align closely with the canonical auxin signaling model (5, 9–14), the mechanism by which class-B ARFs, which constitute the majority of *Arabidopsis* ARF family, function in auxin signaling is less clear. ARF2 protein levels are regulated by ethylene during apical hook development, a process that is dependent on the HOOKLESS1 (HLS1) protein (15). Loss-of-function *arf2* mutant plants exhibit a general increase in stature that is associated with delays in rosette leaf senescence, flowering time, stamen elongation, floral organ abscission (16), and increased seed size (17). Several repressing ARFs, including ARF2, directly recruit TPL/TPR proteins without interacting with Aux/IAA proteins, indicating a different repression mechanism (18). ARF2 recruits TPL/TPR via an EAR domain and a separate RLFGI domain. Mutations in both domains strongly affect ARF2’s repressive function including the inhibition of root hair growth (19). ARF3 is a noncanonical ARF which lacks the PB1 domain. Mutations in the *ARF3/ETT* gene disrupt gynoecium patterning (20, 21). ARF3 can bind to auxin directly through a noncanonical auxin signaling mechanism (22, 23). Class-A and Class-B ARFs share DNA binding motifs in the promoters of some target genes and their competition for binding may play an important role in the auxin-regulated gene expression across land plants (24–29).

Regulation of ARFs at the RNA or protein level is a key component of the auxin-regulated growth response network (30–36). For example, transcription of the class-A ARFs in *Arabidopsis* is regulated by a network of transcriptional repressors (37). The class-B ARFs are regulated by transacting siRNAs, a mechanism that is conserved in all land plants (38). Recently, a small instability (INS) domain within the DBD of B-ARFs in maize and *Physcomitrium patens* was shown to regulate their protein stability and affect auxin responses, revealing a novel layer of protein-level regulation of B-ARFs (29). Additionally, the same minimal region required for MpARF2 degradation was identified in *Marchantia polymorpha*, further indicating that this mechanism is conserved among B-ARFs in plants. Motif swaps analysis further revealed that ARF instability emerged before the divergence of A-ARFs and B-ARFs (39). A recent preprint by de Roij et al. systematically mutated each residue in the MpARF2 INS domain and identified a critical residue required for proteolysis of the B-class MpARF2. They also reported that proteasome-mediated degradation contributes differently to the biological functions of A-class and B-class ARFs in the bryophyte *M. polymorpha* (40). Here, we mutated the reported INS domain of two class-B ARFs in *Arabidopsis*, *ARF2* and *ARF3*, and generated transgenic lines expressing these variants. Native promoter-driven lines carrying a single mutation in the INS domain displayed pleiotropic developmental phenotypes, highlighting the critical roles for ARF2 and ARF3 in plant growth. The persistent ARF2 accumulation also correlates with shorter and branched root hairs. Moreover, these mutant proteins were stabilized and showed disrupted protein ubiquitylation, consistent with a conserved regulatory mechanism among class-B ARFs.

## Results

### Transgenic Lines Expressing Mutated Forms of *EYFP*-*ARF2* Exhibited Pleiotropic Growth Defects.

**Fig. 1.**
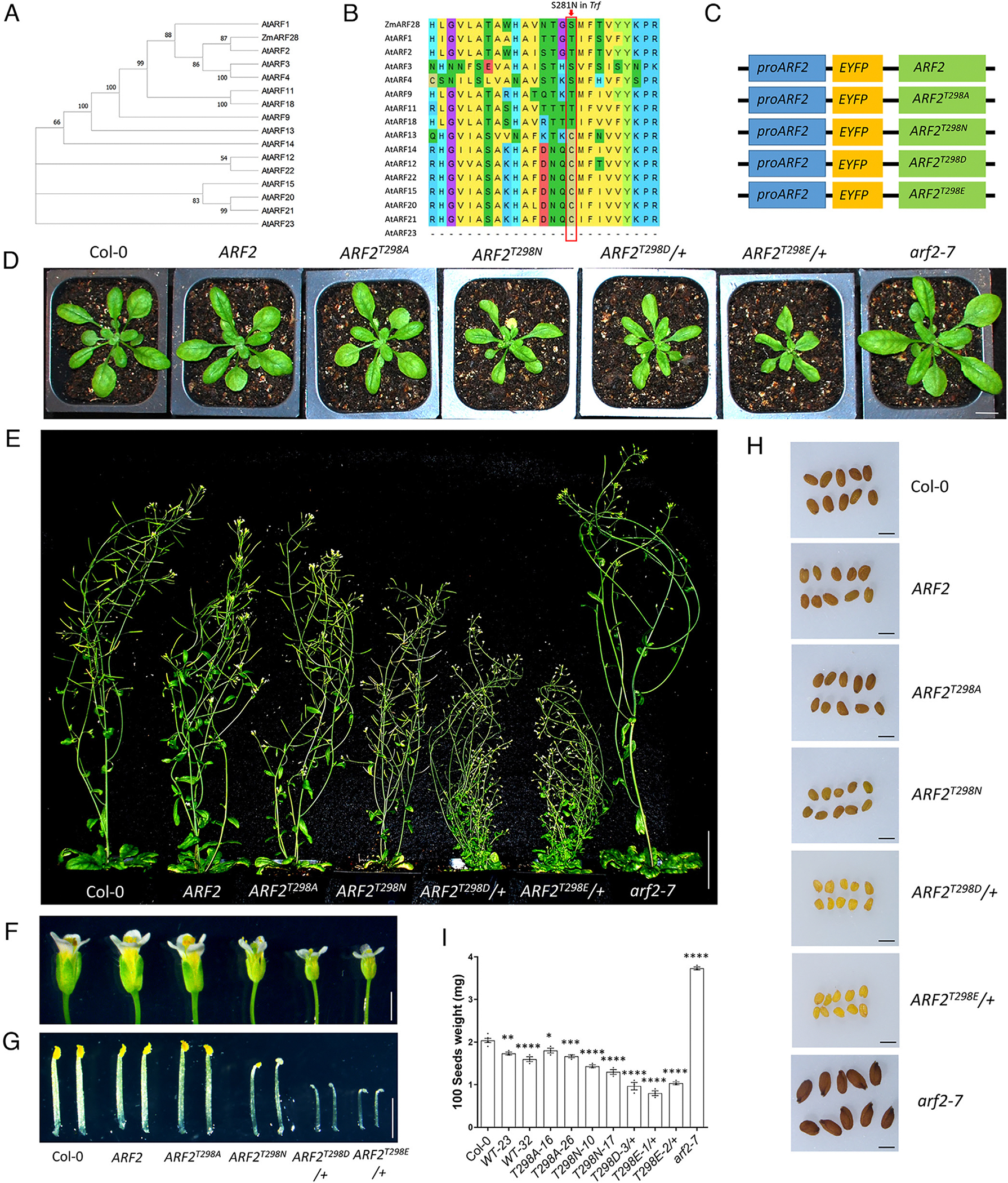
Transgenic lines expressing different mutated forms of *EYFP*-*ARF2* exhibited pleiotropic growth defects. (*A*) Phylogenetic analysis of *Arabidopsis* class-B ARFs with ZmARF28, performed using maximum-likelihood method. (*B*) Multiple sequence alignments of *Arabidopsis* class-B ARFs with ZmARF28 using Clustal W. The mutation site previously identified in maize *Trf* mutant is highlighted in a red box. (*C*) Schematic diagram showing constructs of native promoter-driven EYFP-ARF2 (various mutation forms) transgenic lines in the Col-0 background. (*D*–*H*) Phenotypes of Col-0, different YFP-ARF2 transgenic lines, and *arf2–7* mutant. (*D*) Rosette leaves. (Scale bar, 1 cm.) (*E*) 7-wk plants. (Scale bar, 5 cm.) (*F*) Flowers. (Scale bar, 1 mm.) (*G*) Stamens. (Scale bar, 1 mm.) (*H*) Seeds. (Scale bar, 0.5 mm.) (*I*) Quantification of seed sizes in different lines. One hundred seeds for each genotype were used with three biological replicates. Statistical differences according to one-way ANOVA analysis, * *P* < 0.05, **P < 0.01, ***P < 0.001, ****P < 0.0001. Error bars indicate ±SEM.

To assess whether INS domain function is conserved among class-B ARFs in *Arabidopsis*, we constructed a phylogenetic tree using *ZmARF28* and *Arabidopsis* class-B *ARFs*, identifying *AtARF2* as the ortholog of *ZmARF28*, the candidate gene associated with the *Truffula* (*Trf*) mutant phenotypes (Fig. 1A). Sequence alignment revealed that *AtARF2* and several other *Arabidopsis* class-B *ARFs* share the same amino acid (Serine or Threonine) at the site corresponding to the residue affected in the *Trf* mutant (Fig. 1B). Therefore, *AtARF2* was selected as a representative for further investigation. We substituted the conserved threonine in *AtARF2* with several other amino acids and generated native promoter-driven transgenic lines for each in Col-0 background. These variants include *ARF2*, *ARF2*^*T298A*^*, ARF2*^*T298N*^*, ARF2*^*T298D*^*, and ARF2*^*T298E*^(Fig. 1C and SI Appendix, Fig. S1A).

Among the T1 transformants, no obvious phenotypes were observed in *ARF2* and *ARF2*^*T298A*^ lines. Interestingly, most *ARF2*^*T298D*^ lines and all *ARF2*^*T298E*^ T1 lines exhibited severe pleiotropic phenotypes, including smaller curled rosette leaves, reduced plant height, bushy stature, smaller floral organs, protruding gynoecium, and complete infertility (Fig. 1 D–F and SI Appendix, Fig. S2 A and B). Rosette leaf numbers before flowering in *ARF2*^*T298D*^ and *ARF2*^*T298E*^ were comparable to Col-0 (SI Appendix, Fig. S2C). The *ARF2*^*T298N*^ T1 lines, which correspond to the *Trf* mutation (*ZmARF28*^*S281N*^) in maize, displayed intermediate phenotypes between wild-type (WT) and T298E versions, with compromised fertility defects. Notably, a subset of homozygous *ARF2*^*T298N*^ lines displayed sterility and other similar phenotypes of the ARF2^*T298E*^ T1 lines. In contrast, most homozygous *ARF2* and *ARF2*^*T298A*^ lines appeared largely like Col-0, though a few lines showed slightly reduced stature and shorter siliques (Fig. 1 D–F and SI Appendix, Fig. S2 A–C).

To investigate the sterility phenotype observed in *ARF2*^*T298D/*+^ and *ARF2*^*T298E/*+^ lines, we dissected the flowers and found severe defects in stamen development: Filaments were shortened, anthers markedly reduced in size, and pollen was absent (Fig. 1G). Seed size measurements revealed that these *ARF2* lines produced smaller seeds than the WT, especially in the *ARF2*^*T298N*^, *ARF2*^*T298D/*+^, and *ARF2*^*T298E/*+^ lines (Fig. 1 H–I). In contrast, the *arf2–7* loss-of-function mutant exhibited enlarged rosette leaves, increased stature, longer and thicker inflorescence stems, and produced larger seeds than the WT, similar to what has been observed previously (16, 17) (Fig. 1 D–I). Together, these results suggest that the observed phenotypes in the *ARF2* variants result from accumulation of ARF2 proteins.

### The *ARF2*^*T298D*/+^ and *ARF2*^*T298E*/+^ Mutant Lines Exhibit Root Growth Defects and Auxin Resistant Responses.

**Fig. 2.**
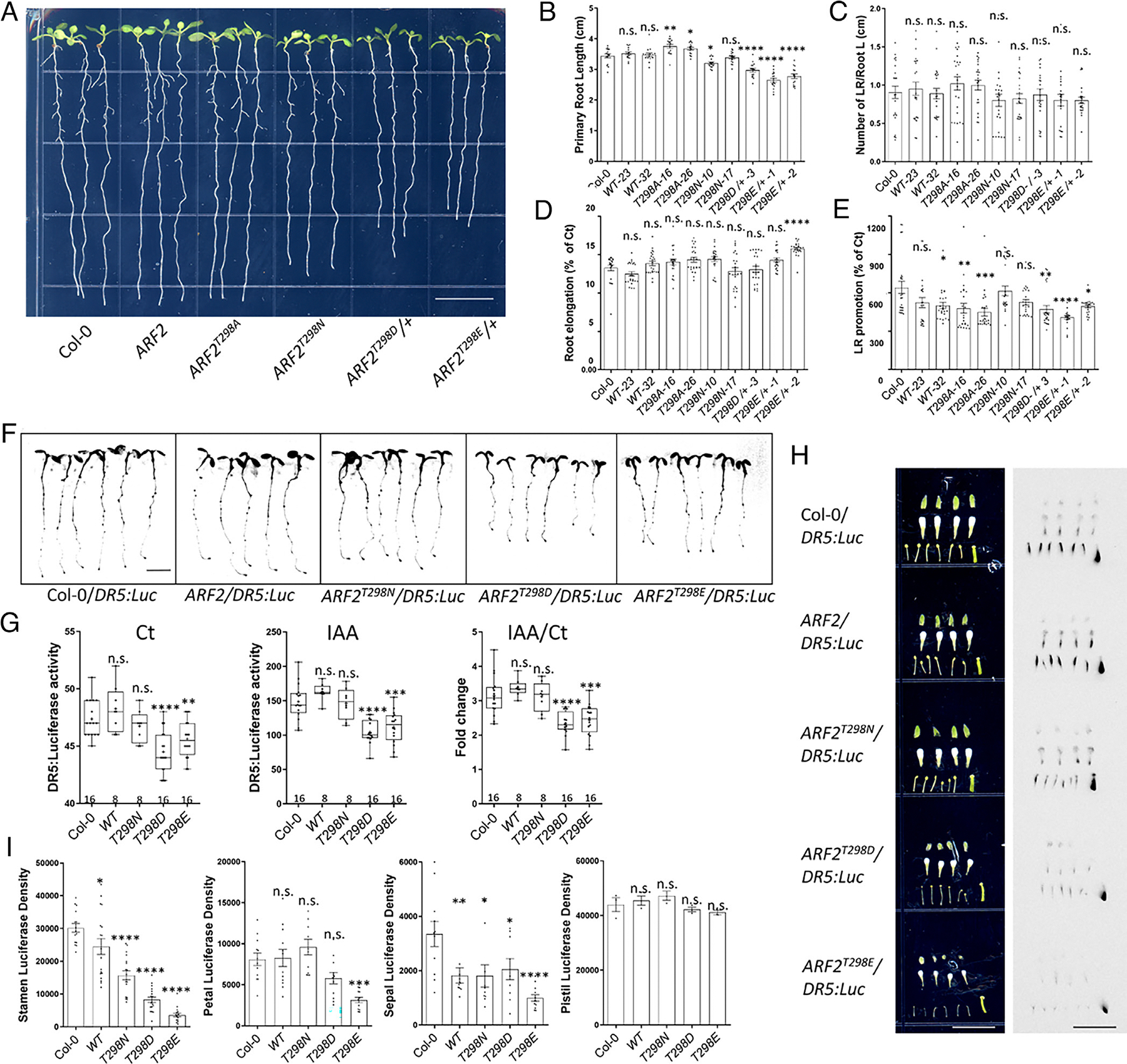
The *ARF2*^*T298D*/+^ and *ARF2*^*T298E*/+^ mutant lines exhibit root growth defects and auxin resistant responses. (*A*) 8-d-old seedlings of Col-0 and various *EYFP-ARF2* transgenic lines. (Scale bar, 1 cm.) (*B*) Quantification of primary root length in Col-0 and *EYFP-ARF2* transgenic lines. (*C*) Quantification of lateral roots density in Col-0 and *EYFP-ARF2* transgenic lines. (*D*) Inhibition of primary root elongation by 200 nM IAA treatment. (*E*) Promotion of lateral roots formation with 200 nM IAA. About 24 seedlings were used in root growth assay in panel (*B*–*E*), statistical differences are according to one-way ANOVA analysis, *P < 0.05, **P < 0.01, ***P < 0.001, ****P < 0.0001. n.s. = not significant. Error bars indicate ±SEM. (*F*) DR5:Luciferase activity in roots of Col-0 and *YFP-ARF2* transgenic lines. (Scale bar, 0.5 cm.) (*G*) Quantification of DR5:Luciferase activity under control and IAA-treated conditions. Box plots represent the median and the first and third quartiles, with whiskers extending to minimum and maximum value; all data points are shown as dots. Statistical differences in these assays are according to Student’s *t* test analysis, *P < 0.05, **P < 0.01, ***P < 0.001, ****P < 0.0001, n.s. = not significant. (*H*) DR5:Luciferase activity in dissected floral tissues of Col-0 and *EYFP-ARF2* transgenic lines. (Scale bar, 0.5 cm.) (*I*) Quantification of DR5:Luciferase activity in different floral organs. Three flowers were used in this assay, statistical differences are according to one-way ANOVA analysis, *P < 0.05, **P < 0.01, ***P < 0.001, ****P < 0.0001. n.s. = not significant. Error bars indicate ±SEM.

We performed root growth assay on *ARF2* lines with or without the addition of IAA. Under normal conditions, *ARF2* lines exhibited primary root lengths comparable to Col-0. *ARF2*^*T298A*^ lines had slightly longer primary roots, whereas *ARF2*^*T298N*^ lines were slightly shorter. Notably, *ARF2*^***T298D/***+^ and *ARF2*^***T298E/***+^ lines showed significantly reduced primary root length compared to Col-0 (Fig. 2 A and B). All lines exhibited similar lateral roots density (Fig. 2C). Upon treatment with 200 nM IAA for 3 d, one *ARF2*^***T298E/***+^ line showed resistance to auxin-induced inhibition of primary root elongation. Moreover, several lines, particularly *ARF2*^***T298D/***+^ and *ARF2*^***T298E/***+^ lines, displayed significant resistance to auxin-induced promotion of lateral roots formation (Fig. 2 D and E).

We crossed these *ARF2* lines with auxin reporter line *DR5:Luciferase* and examined luciferase activity in the F1 generation. In roots, *ARF2* and *ARF2*^*T298N*^ showed luciferase activity comparable to Col-0, whereas *ARF2*^*T298D*^ and *ARF2*^*T298E*^ lines exhibited significantly reduced activity than Col-0 (Fig. 2 F and G). Upon IAA treatment, luciferase activity increased in all lines; however, the fold change in *ARF2*^*T298D*^ and *ARF2*^*T298E*^ lines was notably lower than in the other lines (Fig. 2G). In floral tissues, luciferase activity was reduced in stamens and sepals of all *ARF2* lines. In petals, only *ARF2*^*T298E*^ line showed decreased activity, while no differences were observed in pistil across all lines (Fig. 2 H and I). These results demonstrate that *ARF2*^*T298D*^ and *ARF2*^*T298E*^ lines play negative roles in auxin signaling and show auxin-resistant responses in roots.

### T298D/E Mutations Stabilize the ARF2 Protein and Inhibit Root Hair Development.

**Fig. 3.**
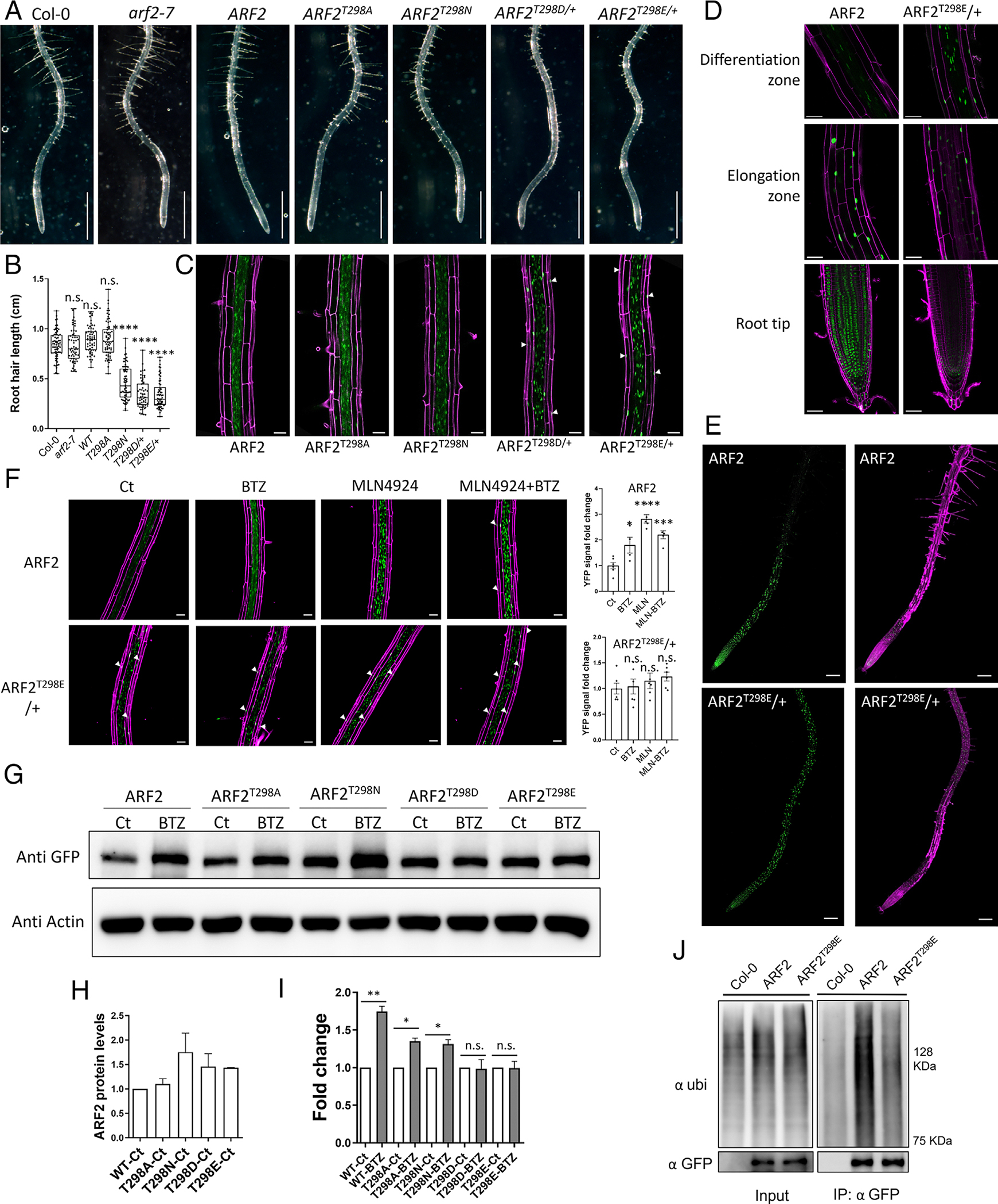
T298D/E mutations stabilize the ARF2 protein and inhibit root hair development. (*A*) Root hair growth in the root tip region of Col-0, *arf2–7*, and *EYFP-ARF2* transgenic lines. (Scale bar, 1 cm.) (*B*) Quantification of root hair length in Col-0, *arf2–7*, and *EYFP-ARF2* transgenic lines. 60 root hairs were measured for each line. Box plots represent the median and the first and third quartiles, with whiskers extending to minimum and maximum value; all data points are shown as dots. Statistical differences are according to one-way ANOVA analysis, ****P < 0.0001. n.s. = not significant. (*C*) EYFP-ARF2 (green) accumulation patterns in different mutant lines in the upper root region. Roots were counterstained with propidium iodide (PI, magenta). White arrowheads indicate signals in epidermal cells. (Scale bar, 50 μm.) (*D*) Accumulation patterns of EYFP-ARF2 (green) in different zones of the roots in *ARF2* and *ARF2*^*T298E/*+^ lines. Samples were stained with PI (magenta). (Scale bar, 50 μm.) (*E*) Accumulation patterns of EYFP-ARF2 (green) correlate with root hair growth in *ARF2* and *ARF2*^*T298E/*+^ lines. Samples were stained with PI (magenta). (Scale bar, 200 μm.) (*F*) Bortezomib (BTZ) and MLN4924 treatments in ARF2 and ARF2^T298E/+^ lines. DMSO, 30 μM BTZ, or 50 μM MLN4924 were treated to 7-d-old seedlings for 6 h and then images were taken. EYFP-ARF2 (green) and PI (magenta). White arrowheads indicate signals in epidermal cells. (Scale bar, 50 μm.) Quantification of YFP signal density in ARF2 and ARF2^T298E/+^ under different treatment conditions is shown in the chart on the right side. About six seedlings for each genotype were used. Statistical differences are according to one-way ANOVA analysis, *P < 0.05, **P < 0.01, ***P < 0.001, ****P < 0.0001. n.s. = not significant. Error bars indicate ±SEM. (*C*–*F*) Representative images from independent lines are shown in these panels (*ARF2–23*, *ARF2*^*T298A*^*-16*, *ARF2*^*T298N*^*-17*, *ARF2*^*T298D*^*-3*, *ARF2*^*T298E*^*-2*). (*G*) Western blot analysis of various EYFP-ARF2 variants in transgenic lines with and without BTZ treatment. DMSO(Ct) or 50 μM BTZ were treated for 6 h. (*H*) Quantification of different EYFP-ARF2 protein levels before BTZ treatment. (*I*) Fold change in EYFP-ARF2 protein levels after BTZ treatment. Fifteen seedlings were used for each sample, and this assay was repeated twice. Statistical differences are according to unpaired *t* test analysis, *P < 0.05, **P < 0.01. n.s. = not significant. Error bars indicate ±SEM. (*J*) Detection of ubiquitylated ARF2 levels in *ARF2* and *ARF2*^*T298E/*+^transgenic lines. Total protein extracts were used as input to detect overall ubiquitylated proteins and EYFP-ARF2. Total proteins were precipitated using GFP-Trap Agrose beads, and protein loading was adjusted to obtain comparable amounts of the EYFP-ARF2 bands. Three biological replicates were performed in these assays.

When examining root hair phenotypes, we found that the *arf2–7* mutant, *ARF2* and *ARF2*^*T298A*^ lines exhibited root hair lengths similar to Col-0. In contrast, *ARF2*^*T298N*^, *ARF2*^*T298D/*+^, and *ARF2*^*T298E/*+^ lines developed shorter and branched root hairs, with *ARF2*^*T298D/*+^ and *ARF2*^*T298E/*+^ lines showing the most pronounced reduction (Fig. 3 A and B). Next, the protein accumulation patterns of different ARF2 variants in roots were analyzed using confocal microscopy. In the root differentiation zone, ARF2, ARF2^T298A^, and ARF2^T298N^ primarily displayed nuclear localization in the stele, with occasional faint signals in endodermal and cortical layers, but little or no detectable signal in epidermal cells. In contrast, ARF2^T298D^ and ARF2^T298E^ showed a distinct pattern with strong accumulation in the stele, endodermal, cortical, and epidermal cells in differentiation zone (Fig. 3C). To distinguish between altered spatial expression and potential defects in protein clearance, we further examined ARF2 and ARF2^T298E^ localization in the meristem, elongation zones, and differentiation zone (onset of root hair formation). Both proteins exhibited clear nuclear signals in epidermal cells in these regions, indicating that ARF2 is normally present in the epidermis at early developmental stages. Notably, a difference emerges in more differentiated epidermal cells at the onset of root hair formation: While the YFP (yellow fluorescent protein) signal is largely absent in wildtype, it persists in the ARF2^T298E^ mutant (Fig. 3D).

Furthermore, we examined ARF2 and ARF2^T298E^ localization from the root tip upward to the differentiation zone. In WT *ARF2* lines, nuclear fluorescence was strong in the root tip and elongation zone, but declined in the differentiation zone, particularly in regions where root hairs initiate, coinciding with the onset of root hair elongation. By contrast, ARF2^T298E^ exhibited sustained nuclear fluorescence from the root tip throughout the differentiation zone, where shorter root hairs were evident by PI staining (Fig. 3E). Together, these results indicate that timely clearance of ARF2 from epidermal cells during differentiation is required for root hair elongation, and that the T298E mutation leads to prolonged ARF2 persistence that inhibits this process. This conclusion is further supported by previous reports showing that root hair–specific expression of ARF2 suppresses root hair growth (19). We propose that the INS domain acts to reduce ARF2 levels in the epidermis, thereby enabling root hair elongation.

We also examined the EYFP-ARF2 localization in stamens of *ARF2* and *ARF2*^*T298E*^ lines. In the *ARF2*^*T298E*^ line, stamen development was arrested at a very early stage, whereas *ARF2* stamens developed normally and produced abundant pollen, as observed in the bright-field images. Consequently, the EYFP-ARF2 fluorescence patterns differed markedly between the two lines (SI Appendix, Fig. S5).

Next, we treated *ARF2* and *ARF2*^*T298E*^ lines with the proteasome inhibitor BTZ and the neddylation inhibitor MLN4924, which inhibits Cullin-RING E3 ligase activity. ARF2 fluorescence increased after BTZ or MLN4924 treatment, and nuclear signals in epidermal cells were observed when both inhibitors were applied. In contrast, ARF2^T298E^ showed no obvious changes under the same treatments (Fig. 3F), indicating that WT ARF2 undergoes CRL-mediated ubiquitylation and proteasome-dependent degradation, while the T298E mutation stabilizes the ARF2 protein by preventing its degradation. Western blot analysis supported this: BTZ treatment increased ARF2 and ARF2^T298A^ protein levels by 1.74- and 1.35-fold, respectively. ARF2^T298N^ showed a moderate 1.3-fold increase. ARF2^T298D^ and ARF2^T298E^ were largely stabilized, reflecting reduced proteasome-dependent degradation (Fig. 3 G–I). Quantification of YFP intensities in different *ARF2* lines under BTZ treatment reinforced this conclusion (SI Appendix, Fig. S3). To further verify that ARF2 protein is ubiquitylated and subsequently degraded, *ARF2* and *ARF2*^*T298E*^ lines were treated with BTZ, EYFP-ARF2 was precipitated using GFP (green fluorescent protein)-Trap beads, and ubiquitylation levels were examined. The results showed a much higher level of ubiquitylation in ARF2 compared to ARF2^T298E^, confirming that T298E mutation disrupts ARF2 ubiquitylation and turnover (Fig. 3J).

### N or E Mutations in the INS Domain of ARF3-mYPet Lines Cause Pleiotropic Growth Defects and Increase Their Protein Stability.

**Fig. 4.**
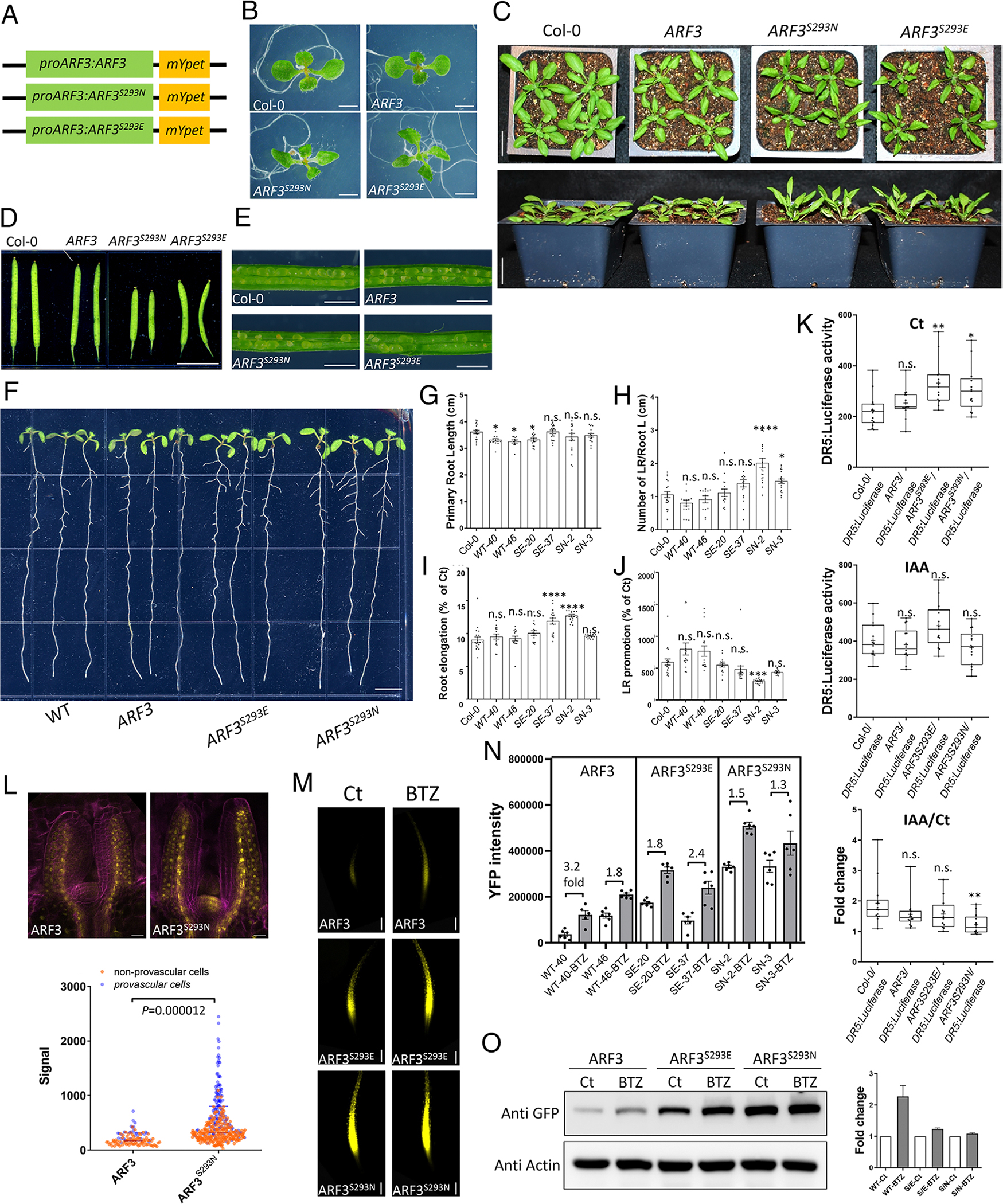
N or E mutations in the “stable domain” of ARF3-mYPet lines cause pleiotropic growth defects and increase their protein stability. (*A*) Schematic diagram of native promoter-driven *ARF3-mYPet* (various mutation forms) constructs in the Col-0 background. (*B*–*F*) Phenotypes of Col-0 and different ARF3-mYPet lines. (*B*) Seedlings. (Scale bar, 2 mm.) (*C*) Top and side views of rosette leaves. (Scale bar, 2 cm.) (*D*) Siliques. (Scale bar, 0.5 cm.) (*E*) Dissect siliques. (Scale bar, 1 mm.) (*F*) Roots. (Scale bar, 0.5 cm.) (*G*) Quantification of primary root length in Col-0 and *ARF3-mYPet* transgenic lines. (*H*) Quantification of lateral roots density in Col-0 and *ARF3-mYPet* transgenic lines. (*I*) Inhibition of root elongation with 200 nM IAA. (*J*) Promotion of lateral roots formation with 200 nM IAA. About 20 seedlings were used for the root growth assays in panel (*G*–*J*). Statistical differences were determined using one-way ANOVA analysis, *P < 0.05, **P < 0.01, ***P < 0.001, ****P < 0.0001. n.s. = not significant. Error bars indicate ± SEM. (*K*) Quantification of DR5:Luciferase activities in Col-0 and *ARF3-mYPet* lines under control and IAA-treated conditions. Box plots represent the median and the first and third quartiles, with whiskers extending to minimum and maximum value; all data points are shown as dots. 14 seedlings in each genotype were used. Statistical differences are according to one-way ANOVA analysis with the same significance notation above. (*L*) Accumulation patterns and quantification of YFP signals in *ARF3* and *ARF3*^*S293N*^ leaves. ARF3-mYpet (yellow) and calcofluor (magenta). (Scale bar, 20 μm.) Four *ARF3* leaves (106 nuclei) and eight *ARF3*^*S293N*^ leaves (361 nuclei) were used for quantification of YFP intensity. Additional images showing similar trends were not included in the quantification. Statistical differences were determined using Welch’s *t* test (unpaired replicates). The *P* values for nonprovascular cells and provascular cells between two genotypes are 0.000351 and 0.000025. (*M*) ARF3-mYPet (yellow) fluorescence in root tips under control and BTZ-treated conditions. DMSO or 50 μM BTZ were treated for 6 h and then images were taken. (Scale bar, 100 μm.) Representative images from one of the analyzed independent lines are shown (*ARF3–46*, *ARF3*^*S293E*^*-20*, *ARF3*^*S293N*^*-2*). (*N*) Quantification of YFP intensity in ARF3 lines with or without BTZ treatment. Six seedlings for each genotype were used. (*O*) Western blot of different versions ARF3-mYPet in transgenic lines, with and without BTZ treatment. DMSO(Ct) or 50 μM BTZ were treated for 6 h. The chart on the right showed quantification results. Fifteen seedlings were used for each sample, assays were repeated twice.

To investigate whether the INS domain is conserved in other class-B ARFs, we cloned the *ARF3* gene and generated two point mutants -*ARF3*^*S293N*^ and*ARF3*^*S293E*^ -under control of the native *ARF3* promoter and tagged with mYPet (Fig. 4A). We obtained 12 homozygous *ARF3* lines, 12 *ARF3*^*S293E*^ lines, and 4 *ARF3*^*S293N*^ lines. All 4 *ARF3*^*S293N*^ lines exhibited down-curled cotyledons/rosette leaves, 6 of 12 *ARF3*^*S293E*^ lines and 2 of 12 *ARF3* lines showed similar leaf phenotypes (Fig. 4 B and C). These stronger lines also exhibited short siliques and reduced fertility, producing fewer seeds (Fig. 4 D and E). All other homozygous *ARF3* lines showed no visible phenotypes. Notably, in a few T1/T2 *ARF3*^*S293N*^ and *ARF3*^*S293E*^ plants, some flowers have five sepals, and these plants were infertile. Dissection revealed severe defects in style development, including unfused styles and the absence of a stigma, which resulted in complete sterility (SI Appendix, Fig. S6). These observations suggest that S293N or S293E substitutions can cause severe growth defects.

Then we examined root phenotypes and performed root growth assay in these *ARF3* lines with or without the addition of IAA. The primary root lengths of *ARF3* and most mutant lines were comparable to Col-0, although *ARF3* and one *ARF3*^*S293E*^ line were slightly shorter. Lateral root density in *ARF3* lines were similar to Col-0, while *ARF3*^*S293E*^ mutant lines showed slightly higher densities than Col-0. Notably, the lateral roots density in *ARF3*^*S293N*^ lines were significantly higher than Col-0 (Fig. 4 F–H and SI Appendix, Fig. S1B). Upon 200 nM IAA treatment, *ARF3*^*S293E*^*-37* and *ARF3*^*S293N*^*-2* lines were resistant to auxin-induced inhibition of primary root elongation (Fig. 4I). In addition, two *ARF3*^*S293N*^ lines showed resistance to auxin-induced promotion of lateral roots formation, with the *ARF3*^*S293N*^*-2* line showing a significant difference compared to Col-0 (Fig. 4J). *DR5:Luciferase* assays in F1 lines revealed that *ARF3* line activity was similar to Col-0, whereas both *ARF3*^*S293E*^ and *ARF3*^*S293N*^ lines displayed significantly elevated basal luciferase levels. Following IAA treatment, luciferase levels in all *DR5:Luciferase* lines increased, but the fold change in *ARF3*^*S293N*^ was significantly lower than in other genotypes (Fig. 4K).

We analyzed the ARF3 localization patterns in different *ARF3* lines in the root tip and upper root. ARF3, ARF3^S293E^, and ARF3^S293N^ exhibited similar accumulation patterns in the roots. In root tips, fluorescence was observed in the cortex, endodermis, and stele, with higher accumulation in the transition zone. In upper roots, all lines localized to the stele region. Notably, ARF3^S293N^ displayed stronger signals than the other two lines (SI Appendix, Fig. S7). Next, to examine whether altered protein accumulation of ARF3 contributes to the observed leaf phenotypes, we analyzed the localization of ARF3 and ARF3^S293N^ on the adaxial and abaxial sides of leaves. Both proteins showed similar distribution, with predominantly stronger signals on the abaxial side. However, despite comparable transcriptional levels between the two lines, ARF3^S293N^ consistently displayed higher fluorescence intensity than WT ARF3, suggesting increased protein accumulation. These results indicate that the leaf curling phenotype is likely associated with elevated ARF3 protein levels (Fig. 4L).

Next, we performed BTZ treatment on the three *ARF* 3 lines. In contrast to the *ARF2* lines, where ARF2^T298E^ was highly stable, all three ARF3 proteins accumulated after BTZ treatment, with the fold changes of 3.2 and 1.8 for two *ARF3* lines; 1.8 and 2.4 for two *ARF3*^*S293E*^ lines; and 1.5 and 1.3 for two *ARF3*^*S293N*^ lines, respectively (Fig. 4 M and N). The results indicate that ARF3^S293E^ and ARF3^S293N^ proteins exhibit increased stability compared with WT ARF3 protein, although both variants still undergo proteasome-dependent degradation, which is also confirmed by western blot analysis (Fig. 4O). Notably, ARF3^S293N^ showed a smaller fold increase after BTZ treatment than ARF3^S293E^, suggesting a relatively stronger stabilization effect. These findings suggest that the INS domain is conserved in the ARF3 protein, however, other residues or domains may contribute to ARF3 protein stability.

## Discussion

Auxin regulates plant growth and development through ARFs, yet how ARF protein levels are precisely controlled in specific tissues is less clear. We show that a conserved instability (INS) domain in class-B ARFs controls protein turnover and spatial accumulation. Mutations that stabilize ARF2 and ARF3 lead to auxin resistance and developmental defects in *Arabidopsis*, including sterility due to anther defects and impaired root hair elongation, indicating a shared regulatory mechanism among class-B ARFs in land plants.

The *ARF2*^*T298D/*+^ and *ARF2*^*T298E/*+^ lines developed shorter and branched root hairs, which is associated with persistent ARF2 accumulation in root epidermal cells (Fig. 3 A–E). These findings demonstrate that regulated degradation of class-B ARFs is required to restrict their activity in specific cell types and reveal posttranslational control of ARF levels as a key mechanism underlying auxin signaling specificity in plants. Interestingly, the root tip signals were stronger in the *ARF2* line than in the *ARF2*^*T298E*^ line (Fig. 3 D and E and SI Appendix, Fig. S4A). When we treated all *ARF2* transgenic lines with BTZ to assess protein stability in the root tip, we found that although ARF2 and ARF2^T298A^ showed higher fluorescence, they were still subject to degradation, as BTZ further increased their signal. In contrast, ARF2^T298D^ and ARF2^T298E^ exhibited weaker root tip signals but were markedly more stable (SI Appendix, Fig. S4B). In addition, MLN treatment produced similar results (SI Appendix, Fig. S4C). These findings suggest that ARF2 is subject to tissue-specific regulation in the root tip, potentially involving a self-regulating mechanism in which ARF2 contributes to its own transcriptional repression. Such regulation may form a negative feedback loop at an additional transcriptional layer in regulation that is crucial for maintaining cellular ARF homeostasis. Elucidating whether such self-regulation occurs will require further investigation. At the same time, additional protein regions or degradation signals may also contribute to ARF2 stability (SI Appendix, Fig. S4 B and C). Using only the DNA-binding domains of ARF2 and ARF2^T298E^ would allow a more direct assessment of how degradation influences protein accumulation patterns in specific tissues, such as root tips, while avoiding complications arising from potential effects on plant growth and development (39).

The small instability (INS) domain within the DBD of class-B ARFs was first identified in maize and *P. patens,* where it was shown to regulate protein stability and affect auxin responses (29). A minimal region within the same region was identified in *M. polymorpha* and shown to be required for MpARF2 degradation (39). The maize dominant *Trf* mutant was identified in an EMS-mutagenized population, which is a S281N mutation in the INS domain. To further explore how amino acid composition within the INS domain affects ARF stability and function, we mutated the conserved threonine residue in the INS domain of *AtARF2* to four amino acids of distinct biochemical properties: alanine (A), asparagine (N), aspartic acid (D), and glutamic acid (E). Transgenic lines expressing these variants revealed that the T298N substitution caused pleiotropic developmental phenotypes, whereas the T298D and T298E resulted in the most severe defects, including small bushy growth, and complete infertility. In contrast, the T298A mutation did not produce obvious phenotypes, suggesting that the presence of a negative charge at this position interferes with INS domain function. Since serine and threonine residues are potential phosphorylation sites, and aspartic acid and glutamic acid are commonly used as phospho-mimic substitutions, these observations raise the possibility that phosphorylation of this conserved S/T may contribute to regulation of ARF2 stability. Phosphorylation-dependent control of protein ubiquitylation and degradation has been widely reported in yeast, mammalian cells, and plants, which plays important roles in cell growth and stimulus-specific responses (41–44). ARF2 has also been shown to be phosphorylated at other sites, affecting its DNA-binding activity (33, 45). Although we did not detect a phosphorylated form of ARF2 by immunoblotting, such modification may be transient and condition dependent. Further investigation will be required to determine whether phosphorylation directly regulates INS domain activity and class-B ARFs turnover. However, the alanine substitution, which represents a nonphosphorylatable mutation, did not lead to reduced protein accumulation compared with the WT protein, suggesting that alternative mechanisms should also be considered. One plausible explanation is that substitutions within the INS domain alter the local conformation or electrostatic properties of the interaction interface with partner proteins, such as E3 ligase adaptors. Consistent with this notion, the corresponding S/T site in maize and *Arabidopsis* is not strongly conserved outside flowering plants, as reported in *P. patens* and *M. polymorpha* (29, 39), indicating that it may not represent a universally conserved regulatory phosphorylation site.

In ARF3 transgenic lines, both S293N or S293E substitutions caused severe developmental phenotypes. Consistent with this, BTZ treatment assays indicated that ARF3^S293E^ and ARF3^S293N^ proteins are morestable than the WT ARF3, although both variants remain subject to proteasome-dependent degradation (Fig. 4 M–O). These findings suggest that additional residues or domains may cooperate with the INS domain to fine-tune ARF3 protein stability. This interpretation is consistent with a recent work, which showed that an A-class ARF (MpARF1) is subject to proteasomal degradation and that its instability reflects contributions from multiple protein regions (40). It is also noteworthy that class-B ARFs are regulated by ancient transacting siRNAs, highlighting the importance of tight control of class-B ARF levels (31, 32, 38). Since the relative levels of class-A and -B ARFs appear to be a key parameter in determining the readout of the auxin signal, ubiquitin–proteasome-dependent degradation of class-B ARFs is likely to be an important aspect of auxin regulation of plant growth and development.

Altogether, our mutational analyses of the INS domain in class-B ARFs in *Arabidopsis* demonstrate that this short motif plays a pivotal and conserved role in controlling protein turnover, uncovering previously unrecognized roles for ARF2 and ARF3 in regulating plant growth and development. Our study further indicates that this posttranslational control operates in a spatially defined manner, restricting class-B ARF accumulation in specific cell types such as the root epidermis. By linking regulated ARF turnover to root hair elongation, this work provides a mechanistic framework for how auxin signaling specificity can be achieved through precise control of ARF protein abundance.

## Methods

### Plant Materials and Growth Conditions.

All *Arabidopsis thaliana* plants used as WT controls and for generating transgenic lines were in Col-0 background. The *arf2–7* mutant is the CS24601 (46). Seeds were surface sterilized with 70% ethanol for 10 min, washed four times with sterile water, and stratified at 4 °C in the dark for 2 d. Stratified seeds were sown on half-strength Murashige and Skoog (MS) media plates with 1% sucrose and 0.8% agar (Sigma-Aldrich, A1296), pH 5.7, and grown at 22 °C under a long-day photoperiod (16L/8D) for phenotypic analyses. Plants for soil growth were cultivated at 22 °C under long-day conditions, while plants used for protoplast preparation were grown in soil at 22 °C with a short-day (8L/16D) photoperiod for 4 wk.

### Molecular Cloning.

To construct various *proARF2:EYFP-ARF2* plasmids, *ARF2*, *ARF2*^*T298A*^,*ARF2*^*T298N*^,*ARF2*^*T298D*^, and *ARF2*^*T298E*^, a 2,018 bp fragment containing the ARF2 promoter was cloned into the multiple cloning site of pMCS:YFP-Gateway (47) to create proARF2:YFP-GW. pENTR-ARF2 (29) was mutagenized using site directed mutagenesis (In-fusion, Takara) with primers listed in SI Appendix, Table S1 to create pENTR-*ARF2*^*T298A*^, pENTR- *ARF2*^*T298N*^, pENTR- *ARF2*^*T298D*^, and pENTR- *ARF2*^*T298E*^. These ARF2 variants were recombined into proARF2:YFP-GW using LR Clonase (Life Technologies) to form *proARF2:EYFP-ARF2* plasmids, *ARF2*, *ARF2*^*T298A*^, *ARF2*^*T298N*^, *ARF2*^*T298D*^, and *ARF2*^*T298E*^. Recombinant plasmids were transformed into Agrobacterium strain GV3101 which was used to transform plants using the floral dip method. Transformants were selected in the presence of 10 μg/mL Basta (phosphinothricin) and lines homozygous for the transgene were identified in subsequent generations.

The *proARF3:ARF3-mYPet* plasmids were assembled using Golden Gate assembly. Two *ARF3* genomic fragments—a 5,557 bp fragment containing the *ARF3* promoter and the first 1,714 bp from the translational start and a downstream 1,417 bp fragment of *ARF3* genomic DNA—with flanking BsaI restriction sites were subcloned into pMiniT2 (New England Biolabs). Different versions of the instability domain within the *ARF3* DBD domain (*ARF3*, *ARF3*^*S293N*^, and *ARF3*^*S293E*^) were synthesized as double-stranded oligos with the appropriate overhangs. These three fragments plus a plasmid containing a monomeric bright YFP gene, *mYPet*, fragment were assembled into the pMP2200 Golden Gate destination vector to generate the final constructs. Primers used for gene cloning are listed in SI Appendix, Table S1.

### Sequences Alignment and Phylogenetic Tree.

Fifteen class-B *ARFs* from *Arabidopsis* and maize *ARF28* were aligned using Clustal W. Phylogenetic trees were inferred in MEGA-X using the maximum-likelihood method (48). The bootstrap consensus tree inferred from 1,000 replicates is taken to represent the evolutionary history of the taxa analyzed, and branches corresponding to partitions reproduced in less than 50% bootstrap replicates are collapsed. The bootstrap values (%) are shown next to the branches (49).

### Root Growth Assay and Root Hair Characterization.

Stratified seeds were sown onto ^1/2^ MS medium supplemented with 1% sucrose and incubated vertically in a growth chamber at 22 °C. Five-day-old seedlings were transferred to 120 mm square plates containing the same medium, with or without 200 nM IAA. For each genotype, seedlings were arranged on the top row of one plate and the bottom row on a second plate, which was placed in a different part of the growth chamber. After marking the root tips positions, the plates were scanned with Epson V600 flatbed scanners. The plates were scanned again after 72 h, and root growth was measured using imageJ. The numbers of lateral roots were counted using a dissecting microscope.

Root hair length was measured as previously described with modifications (50). Hairs within the same root region (0.78 mm from the hair maturation region) were selected for measurement. For each root, five hairs protruding perpendicularly from each side of the root, 10 hairs in total, were quantified with ImageJ.

### Luciferase Activity Assay.

*ARF2* and *ARF3 DR5:Luciferase* F1 reporter lines were used for luciferase activity measurement. For imaging, 5-d-old seedlings or dissected floral tissues were treated with 1 mM D-Luciferin (Potassium Salt, Gold Biotechnology, LUCK-1G, in 1× PBS) in the dark, and then imaged using the western blot imaging system (ImageQuant LAS 4000 mini). For the quantitative auxin response assay, 7-d-old seedlings were dissected, and the roots were transferred into 96-well plates containing 1/2 MS medium. The 1/2 MS medium was then replaced with 1 mM D-Luciferin, and the samples were incubated for 10 min in the dark. Luciferase luminescence was measured using BioTek Synergy H1 microplate reader. Next, the D-Luciferin solution was removed and replaced with 5 μM IAA (in 1/2 MS medium) for a 30-min treatment. After incubation, the IAA-containing medium was removed and luciferase luminescence was measured again. Seven seedlings in each genotype were used in this assay.

### RNA Extraction and RT-qPCR Analysis.

RNA extraction and RT-qPCR assays were performed as described previously (51), with minor modifications. Seedlings, roots, and flowers were collected and used for total RNA isolation using Qiagen RNeasy Plant Mini kit. The quality of the total RNA was assessed using a NanoDrop spectrophotometer. Two micrograms of RNA were used for RT-PCR using Thermo Maxima H Minus master mix. qRT-PCR was performed using CFX Opus 384 Real-Time PCR System (Bio-Rad) with SsoAdvanced SYBR mix (Bio-Rad). Primers used for qRT-PCR are listed in SI Appendix, Table S1.

### Microscopy Imaging.

Seven-day-old *ARF2* and *ARF3* transgenic seedlings were stained with PI, mounted with water, and observed with a Zeiss LSM 880 inverted microscope. Tile-scan mode was used for capturing images of root tip through upper root. All images in the same assay were taken using the same microscope settings. Stamens were dissected from the first opened flower and imaged with a Keyence microscope. Root tips and upper roots of *ARF2* and *ARF3* transgenic lines treated with BTZ were also viewed with a Keyence microscope using a 20× objective.

For leaves imaging in *ARF3* transgenic lines, after cotyledon removal with a double-edge razor, 4-d-old seedlings were fixed in 4% formaldehyde (Electron Microscopy Sciences 15710) and 0.4% DMSO in 1× PBS for 40 to 80 min including 10 min under vacuum. Seedlings were washed thrice for 1 to 5 min with 1× PBS and incubated with ClearSee-α twice for 2 d (52). Cleared seedlings were stained with 0.1% Calcofluor White M2R in ClearSee-α for 30 min and rinsed in ClearSee-α for another 30 to 90 min (53). Samples were imaged with a Leica Stellaris 5 with HyD detectors microscope using the 20×/0.75NA objective. mYPet was excited with the 514 nm laser and 525 to 626 nm detection window, and Calcofluor was excited with the 405 nm laser and 414 to 488 nm detection window.

### Western Blotting and Ubiquitylation Assay.

Western blotting and Ubiquitylation assays were performed as described previously (51), with minor modifications. Seven-day-old seedlings of *ARF2* transgenic lines were treated with DMSO (dimethyl sulphoxide) and 50 μM BTZ for 6 h. Then, seedlings were collected and extracted using protein extraction buffer [50 mM Tris-HCl (pH7.5), 150 mM NaCl, 5 mM EDTA, 1 mM PMSF, 1 mM dithiothreitol, protease inhibitor cocktail (Roche, 11836170001) and 1% Triton X-100] with 30 μM BTZ. Extracted proteins were boiled in SDS protein loading buffer for 5 min. Immunoblots were detected with an anti-GFP antibody (1:2,000; Roche, 11814460001).

In ubiquitylation assay, 14-d-old seedlings in different genotypes were treated with 50 μM BTZ overnight. Seedlings were then collected and extracted using 1 mL protein extraction buffer. Move 100 μL total protein for input, and add 25 μL equilibrated GFP-Trap magnetic beads (Bulldog Bio, GMA020) to 900 μL protein. After 2 h rotated incubation at 4 °C, the beads were gathered using a magnetic stand, washed three times with protein extraction buffer and then eluted by boiling the beads in SDS protein loading buffer for 5 min. Immunoblots were detected with an anti-GFP antibody (1:2,000; Roche, 11814460001) and antiubiquitin antibody (Cell Signaling Tech, 3936T, 1:1,000).

## Supplementary Material

Supplementary Material

This article contains supporting information online at https://www.pnas.org/lookup/suppl/doi:10.1073/pnas.2537963123/-/DCSupplemental.

## Data Availability

All study data are included in the article and/or SI Appendix.

## Significance

Auxin has a broad role in the regulation of many aspects of plant growth and development, including aspects that are important for food production. Auxin acts via transcription factors, called AUXIN RESPONSE FACTORS (ARFs). The ARFs fall into subclasses including class-A and -B that act as transcriptional activators and repressors, respectively. Studies show that the relative levels of these two ARF classes affect the magnitude of the auxin response. In this work, we show that class-B ARF levels are regulated by the ubiquitin–proteasome system. Class-B ARF stabilization results in a dramatic decrease in auxin response. Notably, loss of class-B ARFs stimulates plant growth, suggesting that manipulation of this system might have a role in crop optimization.
