# Supplementary material for: Mutational analyses of an instability domain reveal its conserved role in the regulation of class-B ARF levels in Arabidopsis
Source: Proc Natl Acad Sci U S A. Author manuscript; Available in PMC 2026 Jul 13. (PMC13250565; doi:10.1073/pnas.2537963123)
Supplement: Supplementary Material [file NIHMS2185324-supplement-Supplementary_Material.pdf]

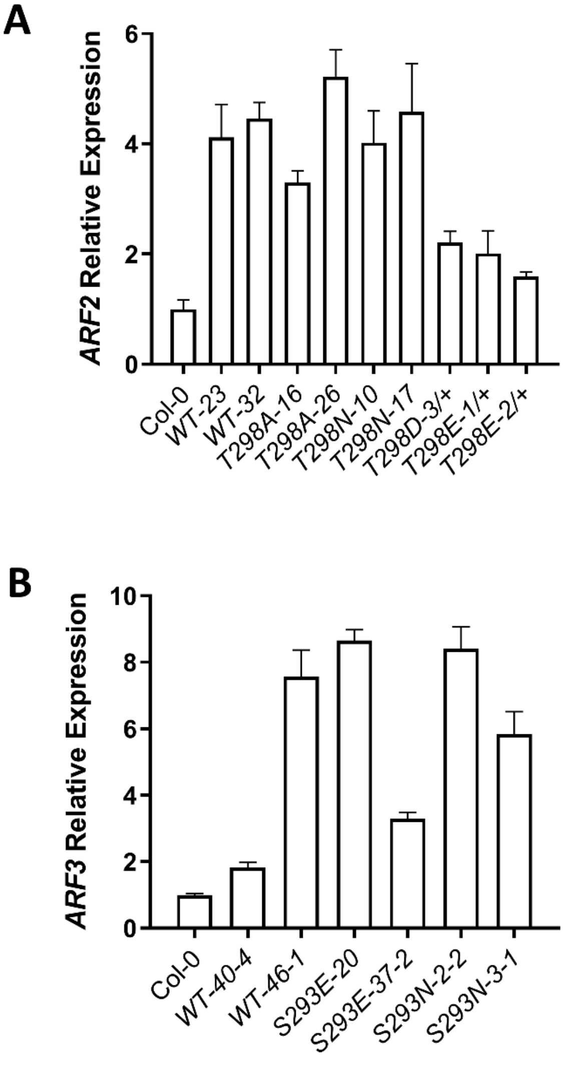

Fig. S1. Transcriptional levels of different ARF2 and ARF3 transgenic lines.

(A) Transcriptional levels in different ARF2 lines. (B) Transcriptional levels in different ARF3 lines. Ten seedlings in each line were used in these assays. Data are represented as mean ± SEM.

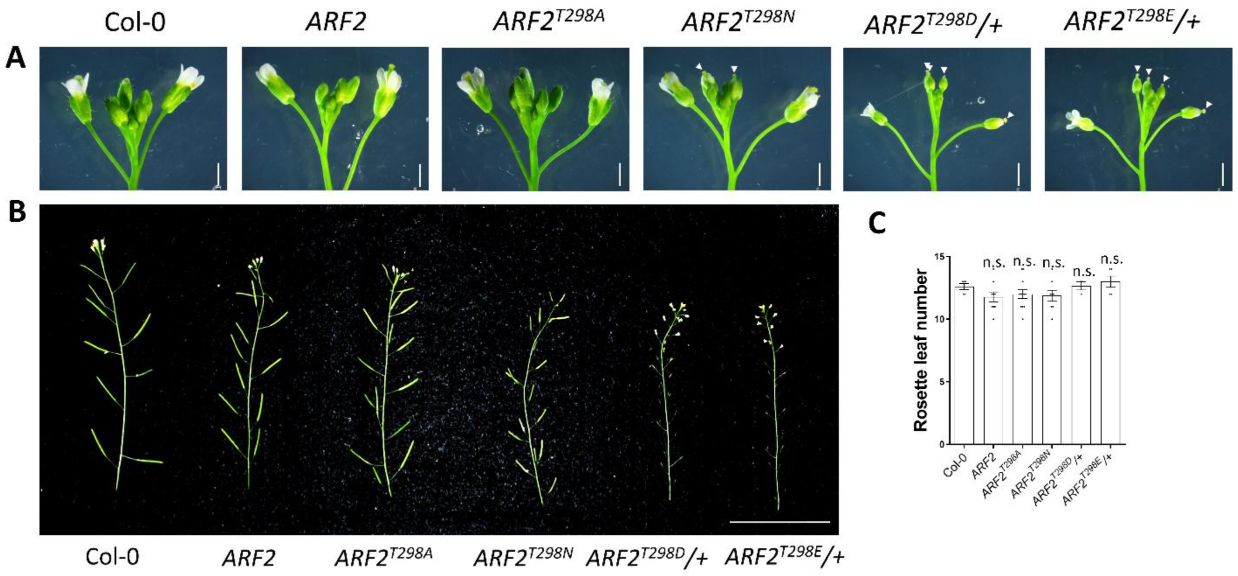

Fig. S2. Different *EYFP-ARF2* transgenic lines exhibited pleiotropic growth defects.

(A) Inflorescences. Scale bar=1mm. (B) Inflorescence stems. Scale bar=5cm. (C) Quantification of rosette leaves number. Statistical differences were determined using one-way ANOVA analysis, **P* < 0.05, n.s. = not significant. Error bars indicate ±SEM.

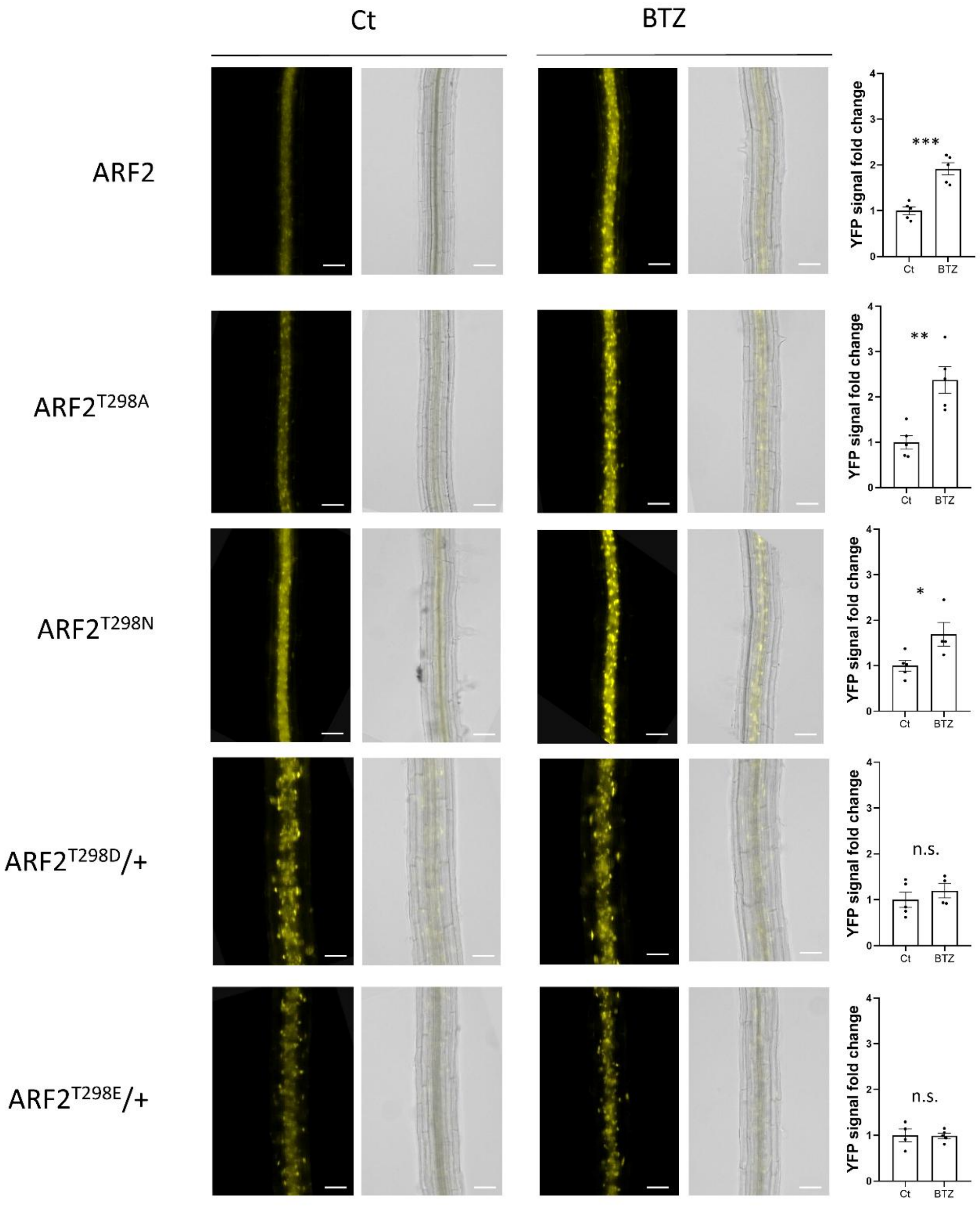

Fig. S3. BTZ treatment in different mYPet-ARF2s transgenic lines.

DMSO or 50 μM BTZ were treated for six hours and then images were taken. EYFP-ARF2 (yellow) and bright-field showing root structures. Scale bar=50 μm. Quantification of YFP signal intensity in *ARF2* lines under different treatment conditions are shown in the chart on the right. About five seedlings for each genotype were used. Statistical differences is according to Student's T-test analysis, *$P < 0.05$, **$P < 0.01$, ***$P < 0.001$. n.s. = not significant. Error bars indicate ±SEM.

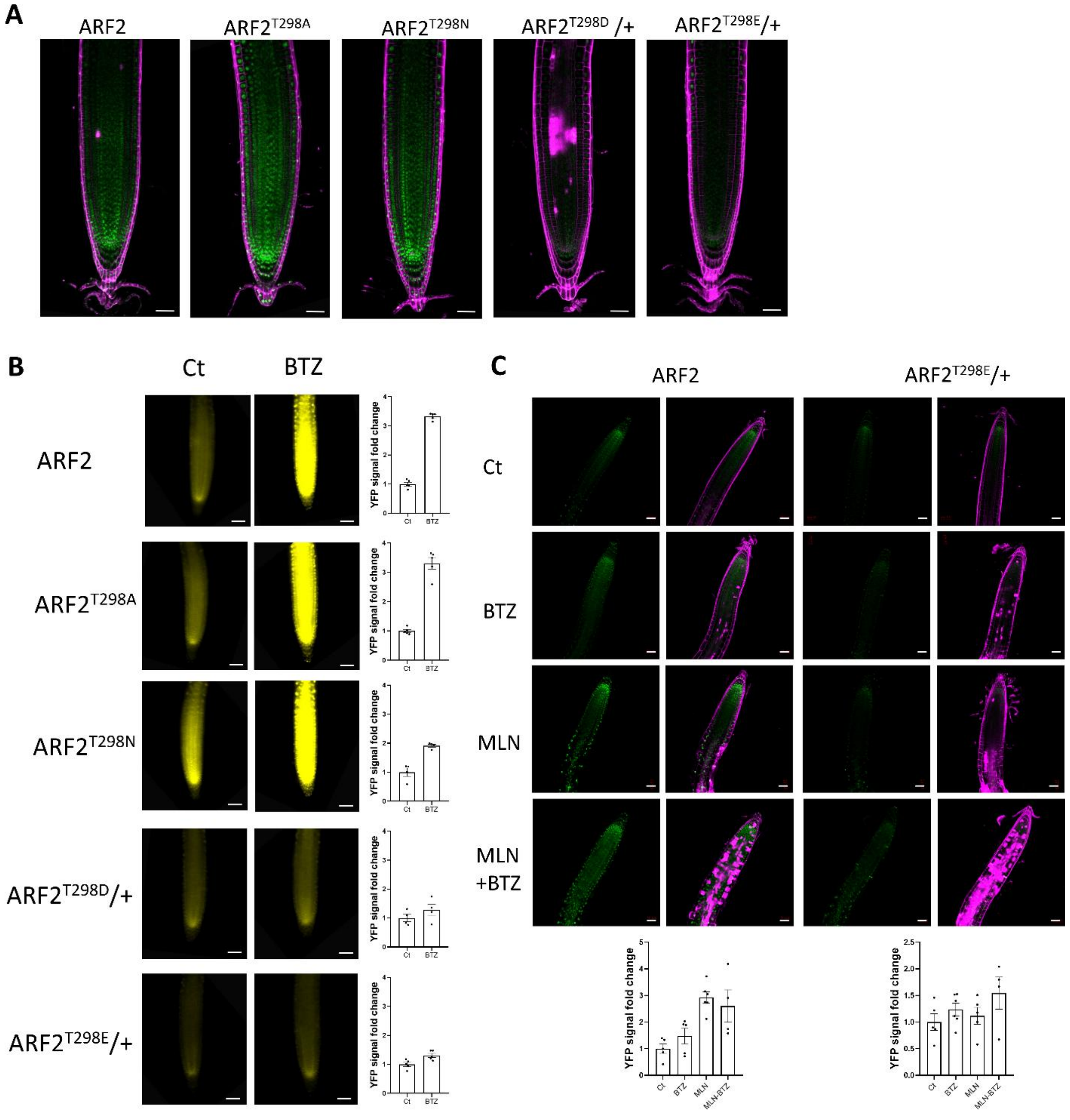

Fig. S4. ARF2 proteins are stabilized in *ARF2^{T298D}*/+ and *ARF2^{T298E}*/+ mutant root tips.

(A) EYFP-ARF2 (green) accumulation patterns in different *ARF2* lines in the root tip. Roots were counterstained with propidium iodide (magenta). Scale bar=50 µm. (B) EYFP-ARF2 (yellow) in different lines under control and BTZ treated conditions. DMSO or 50 µM BTZ were treated for six hours and then images were taken. Scale bar=50 µm. Quantification of YFP signal intensity are shown in the chart on the right. Approximately five seedlings per genotype were analyzed. Error bars indicate ±SEM. (C) BTZ and

MLN4924 treatments in ARF2 and ARF2^{T298E}/+ lines in root tips. DMSO, 30 μM BTZ or 50 μM MLN4924 were treated to 7-day-old seedlings for six hours and then images were taken. EYFP-ARF2 (green) and propidium iodide (magenta). Scale bar=50 μm. Quantification of YFP signal density in ARF2 and ARF2^{T298E}/+ under different treatment conditions is shown in the chart below. About six seedlings for each genotype were used. Error bars indicate ±SEM.

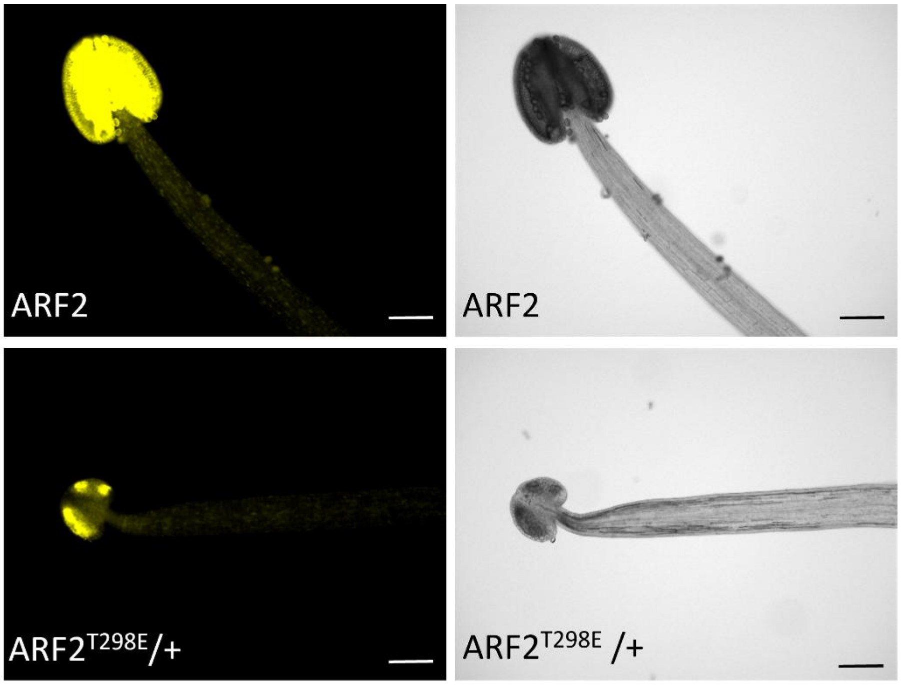

Fig. S5. EYFP-ARF2 pattern in the stamens of *ARF2* and *ARF2^{T298E}* lines.

EYFP-ARF2 (yellow) and bright-field showing stamen structures. Scale bar=1 mm.

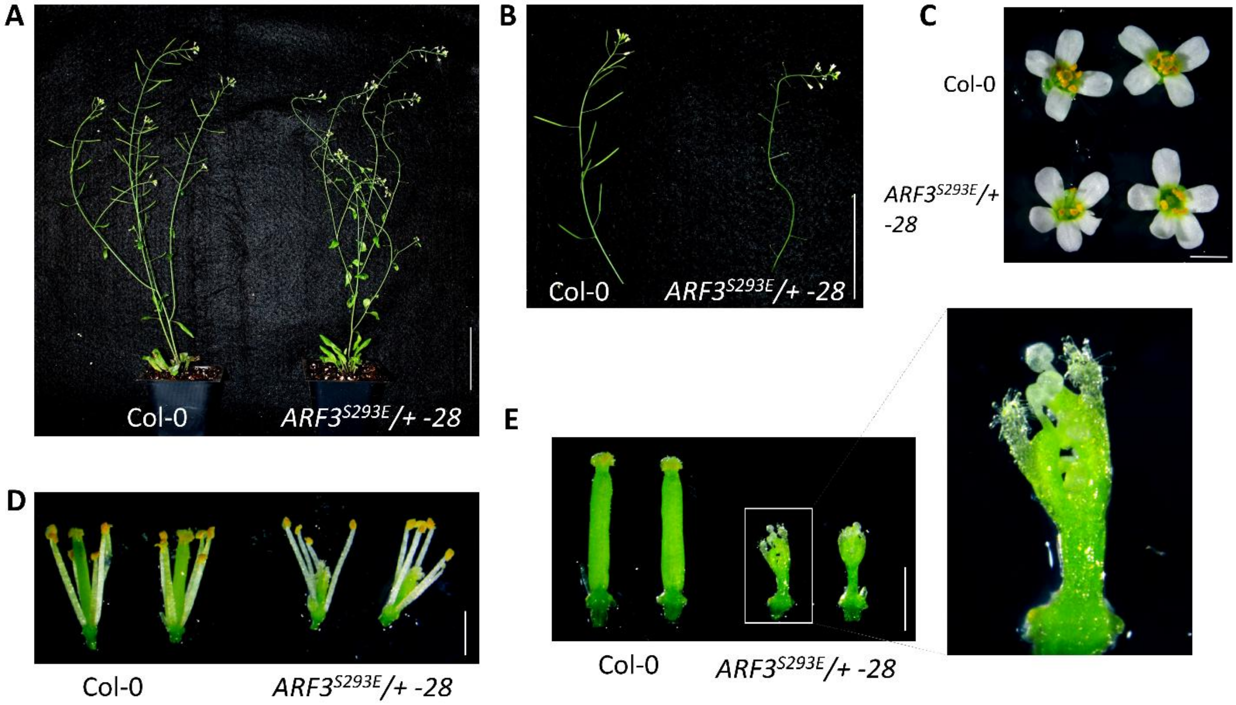

Fig. S6. A few ARF3^{S293E} and ARF3^{S293N} lines showed dramatic phenotypes.

(A) Adult plants. Scale bar=5cm. (B) Inflorescence stems. Scale bar=5cm. (C) Flowers. Scale bar=0.5mm. (D) Flowers (sepals and petals removed). Scale bar=1mm. (E) Pistils. Scale bar=1mm.

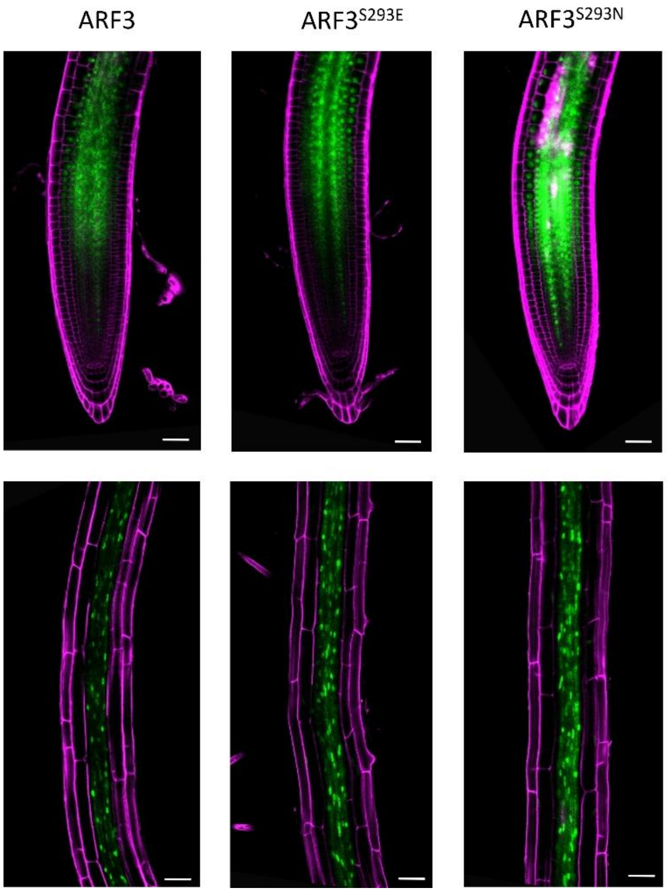

Fig.S7. Accumulation patterns of different ARF3 mutation versions in root tip and upper root tissues.

ARF3-mYPet (green) and propidium iodide (magenta). Scale bar=50 µm. Representative images from independent lines are shown (*ARF3-46*, *ARF3*^{S293E}*-20*, *ARF3*^{S293N}*-2*).

Supplemental Table S1. List of primers used in this study.

Name	Sequence	Purpose
ARF2pro -2006 EcoRI FW	GAATTCGGAATGGCCGAATTACAG TCGA	For cloning the ARF2 promoter into pMCS-YFP-GW
ARF2pro -2006 XhoI RV	CTCGAGACCTTCCGAAGCTCAGAT CTG	For cloning the ARF2 promoter into pMCS-YFP-GW
ARF2_Te2_F1	gcatgccatttcaacagggaatatgtttacagtcta ctaca	For Mutating T to N in ARF2 - Te2 Mutation
ARF2_Te2_R1	tgtagtagactgtaaacatattccctgttgaaatgg catgc	For Mutating T to N in ARF2 - Te2 Mutation
ARF2_T2A_F1	gcatgccatttcaacaggggctatgtttacagtcta cta	For Mutating Te2 T to A in ARF2
ARF2_T2A_R1	tagtagactgtaaacatagcccctgttgaaatggc atgc	For Mutating Te2 T to A in ARF2
ARF2_T298E_F	catggcatgccatttcaacaggggaaatgtttaca gtctactacaaacc	For mutating T298E in AtARF2
ARF2_T298E_R	ggtttgtagtagactgtaaacatttcccctgttgaaa tggcatgccatg	For mutating T298E in AtARF2
ARF2_T298D_F	catggcatgccatttcaacaggggatatgtttaca gtctactacaaac	For mutating T298D in AtARF2
ARF2_T298D_R	gtttgtagtagactgtaaacatatcccctgttgaaat ggcatgccatg	For mutating T298D in AtARF2
mYPet-iF	GGACGACGGCAACTACAAGA	ARF2 lines genotyping
ARF2-Trf-R	tcccaacgattgtgccagtaa	ARF2 lines genotyping
proETT-ggF	CGGTctcTGGagCTGACACAACTGA TTAAAGC	ARF3- 5' cloning
ETTe6-ggR	GggtcTcTaGCgTGAGCTACTTCAGA GAAATTGTTGTG	ARF3- 5' cloning
ETTe6i-ggF	GCggTctCTACAACCCCAAGTAAGC CCC	ARF3- 3' cloning
ETTns-ggR	GggtCtcTCgAaAGAGCAATGTCTAG CAACATGTCTC	ARF3- 3' cloning
ETTsdwt-For	CGCTATATCGACCCATAGCGTTTT CAGCATTTCCT	For cloning AtARF3
ETTsdwt-Rev	TTGTAGGAAATGCTGAAAACGCTA TGGGTCGATAT	For cloning AtARF3
ETTsdsn-For	CGCTATATCGACCCATAaCGTTTT CAGCATTTCCT	For mutating S293N in AtARF3
ETTsdsn-Rev	TTGTAGGAAATGCTGAAAACGtTAT GGGTCGATAT	For mutating S293N in AtARF3
ETTsdse-For	CGCTATATCGACCCATgaaGTTTTC AGCATTTCCT	For mutating S293E in AtARF3
ETTsdse-Rev	TTGTAGGAAATGCTGAAAACttcAT GGGTCGATAT	For mutating S293E in AtARF3
ARF3-seqF	GCGACTGGGAGTTAGAAGAGC	ARF3 lines genotyping
EGFP-N-REV	CGTCGCCGTCCAGCTCGACCA	ARF3 lines genotyping
ARF2-q-For	TGCGACAACAAGGAAACG	qRT-PCR
ARF2-q-Rev	CGGAACAATAAACTCAGATGGG	qRT-PCR

ARF3-q-For	CAAGAGAAGCAGGATTGGC	qRT-PCR
ARF3-q-Rev	ATCGGTTATTGAGTAGGCGG	qRT-PCR
